# Novel phenotypes of prediabetes?

**DOI:** 10.1007/s00125-016-4015-3

**Published:** 2016-06-25

**Authors:** Hans-Ulrich Häring

**Affiliations:** 1 Department of Internal Medicine IV, Division of Endocrinology, Diabetology, Angiology, Nephrology and Clinical Chemistry, University of Tübingen, Otfried-Müller-Str. 10, 72076 Tübingen, Germany; 2 Institute of Diabetes Research and Metabolic Diseases (IDM), University of Tübingen, Tübingen, Germany; 3 German Center for Diabetes Research (DZD), Neuherberg, Germany

**Keywords:** Brain insulin resistance, Insulin, Liver fat, Phenotype, Prediabetes, Review, Secretion, Sensitivity

## Abstract

This article describes phenotypes observed in a prediabetic population (i.e. a population with increased risk for type 2 diabetes) from data collected at the University hospital of Tübingen. We discuss the impact of genetic variation on insulin secretion, in particular the effect on compensatory hypersecretion, and the incretin-resistant phenotype of carriers of the gene variant *TCF7L2* is described. Imaging studies used to characterise subphenotypes of fat distribution, metabolically healthy obesity and metabolically unhealthy obesity are described. Also discussed are ectopic fat stores in liver and pancreas that determine the phenotype of metabolically healthy and unhealthy fatty liver and the recently recognised phenotype of fatty pancreas. The metabolic impact of perivascular adipose tissue and pancreatic fat is discussed. The role of hepatokines, particularly that of fetuin-A, in the crosstalk between these organs is described. Finally, the role of brain insulin resistance in the development of the different prediabetes phenotypes is discussed.

## Introduction

The global increase in type 2 diabetes prevalence over recent decades puts a heavy health and socioeconomic burden on society. Lifestyle intervention with increased physical activity and a healthy diet is considered to be generally effective in preventing the development of diabetes [1–4]. Unfortunately, the prevention studies carried out so far have shown that a substantial number of prediabetic individuals (that is, those with increased risk for type 2 diabetes) seem to be non-responders to lifestyle interventions; the number needed to treat amounted to 7 in the Finnish Diabetes Prevention Study (DPS) and the US Diabetes Prevention Program (DPP) [1, 2]. Based on these studies and on pathophysiology-based studies in our population [5–7], it appears that even those individuals who are able to reduce their body fat mass adequately show a lack of improvement in hyperglycaemia and insulin resistance. To improve the efficacy of lifestyle intervention programmes, a precise understanding of the pathophysiology and the different phenotypes of the prediabetic population is needed.

**Fig. 1:**
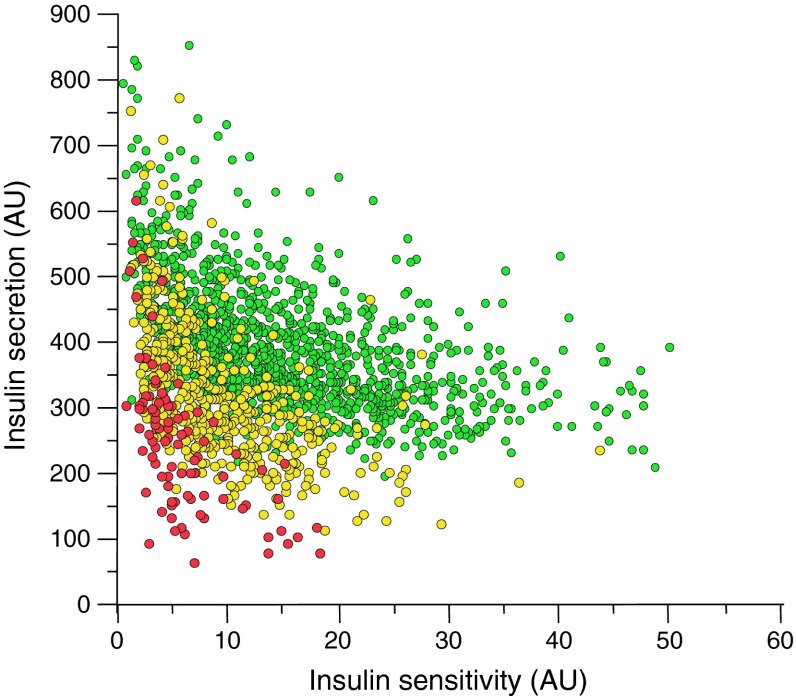
Insulin sensitivity and insulin secretion of participants of the Tübingen Family Study. Insulin sensitivity is estimated from the OGTT (Matsuda–deFronzo index). Insulin secretion is estimated from the OGTT (AUC for C-peptide/glucose). Green circles, NGT; yellow circles, IGT; red circles, type 2 diabetes. AU, arbitrary units

There are several prediabetes cohorts throughout the world [8–13] that provide extensive information about the natural history of disease progression from a prediabetic state to overt type 2 diabetes. Eighteen years ago at the University Hospital of Tübingen we started to build up a similar cohort, which we named the Tübingen Family Study (TÜF). Currently, the cohort comprises more than 3000 individuals from whom we have obtained values for insulin sensitivity and insulin secretion. The distribution of insulin sensitivity and insulin secretion in our cohort (Fig. 1) largely reflects the results from other groups worldwide [8–13]. It is evident that people with high insulin sensitivity very seldom display disturbed glucose tolerance. At higher values of insulin resistance individuals remain glucose tolerant only if their pancreas is able to react with a compensatory hypersecretion of insulin. Partial compensatory hypersecretion characterises individuals within the range of impaired glucose tolerance, while people with the lowest values of insulin secretion often show overt type 2 diabetes. In about 400 participants of the TÜF study, we are now studying the acute and long-term effects on insulin resistance and secretion of a lifestyle intervention programme. We found that there was a significant improvement in insulin resistance after 1 year. However, in follow-up studies after 2 years and 8 years this benefit was not only lost but also, in contrast, increased insulin resistance developed over the years (H-U Häring, unpublished data). These findings are in agreement with those of other earlier studies [8–13]. The stepwise progression towards type 2 diabetes involves increasing insulin resistance, although it is well established that only when combined with a simultaneous loss of compensatory hypersecretion of insulin does this lead to diabetes [8–13]. The major questions are therefore: what pushes people from an insulin-sensitive status to one of insulin resistance and what underlies the ability of an individual to respond with compensatory insulin hypersecretion?

## Compensatory hypersecretion of insulin: influence of genetic variation

Using our database, we assessed the extent to which genetic variation determines the ability to produce compensatory hypersecretion of insulin. A large number of type 2 diabetes loci are known today [14–16] and we studied genotype–phenotype associations for the strongest type 2 diabetes genes [17–41].

We indeed found associations between many of the genetic variants and different features of insulin secretion [17–39]. Several of the gene variants showed interaction with other gene variants, and the degree of the effects was dependent on ligands that induce insulin secretion, such as incretins or fatty acids [17–25]. However, after quantifying the effect of these gene variants and testing for additive effects between variants and genotype–ligand interaction, it appears that the effects on insulin secretion are very small and often non-significant in insulin-sensitive individuals [30, 31]. In insulin-resistant individuals, who have higher insulin levels due to the compensatory effort of the pancreatic beta cell, the reduction in insulin secretion in association with these gene variants is more pronounced and significant [30, 31]. The quantitative effect, however, remains quite small, reaching only 15–20% of the compensatory response observed in individuals with normal glucose tolerance (NGT) vs impaired glucose tolerance (IGT) vs diabetes. This suggests that the type 2 diabetes genes known so far have only minor effects on insulin secretion and that variation in these genes does not contribute much to the large difference in compensatory hypersecretion of insulin that is seen when NGT, IGT and diabetic individuals are compared (Fig. 1).

### *TCF7L2* variants

**Fig. 2:**
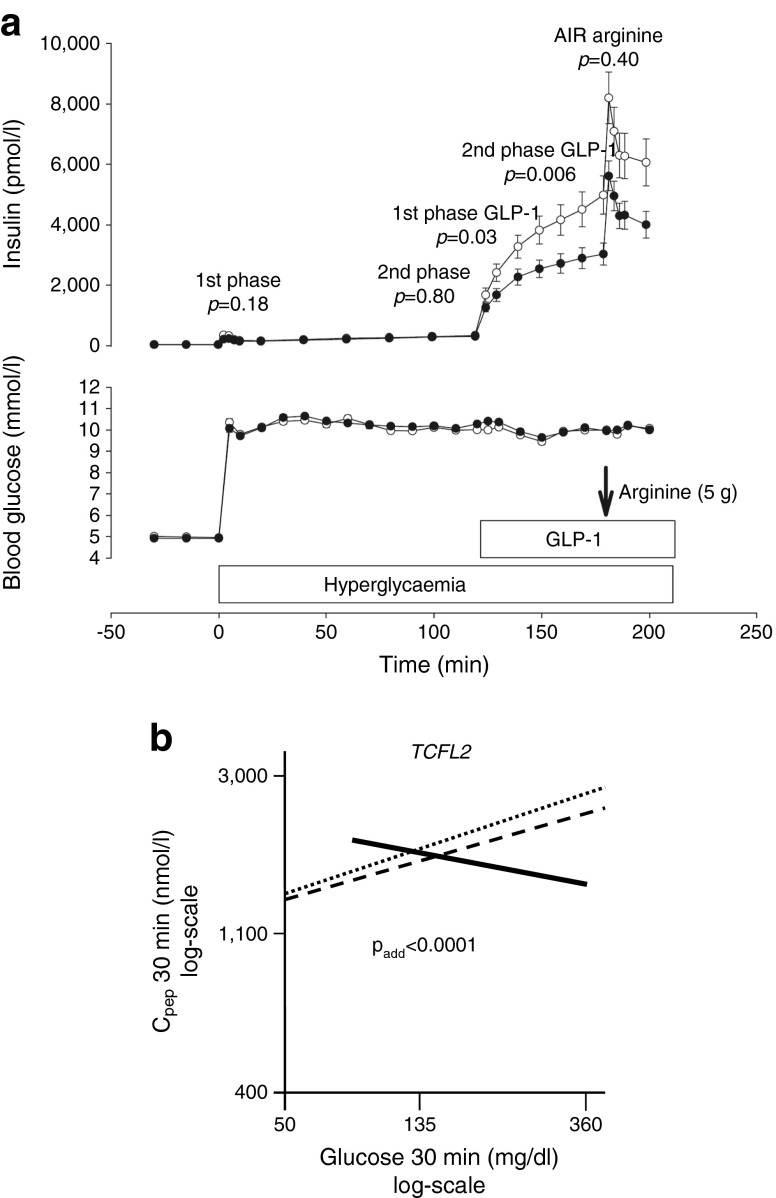
(**a**) Associations between the genotypes of rs7903146 polymorphism in *TCF7L2* with insulin secretion during a hyperglycaemic clamp in 73 German individuals. White circles, CC; black circles, CT and TT. AIR, acute insulin response. The *p* values are for comparison between the genotypes for the first and second phases of glucose-induced insulin secretion, first and second phases of GLP-1-induced insulin secretion and acute insulin secretory response to arginine; figure reproduced with permission from [20]. (**b**) Association between C-peptide levels at 30 min of the OGTT and glucose levels at 30 min during the OGTT by *TCF7L2* SNP rs7903146. Regression lines are shown. Dotted line, CC; dashed line, CT; solid line TT genotype of *TCF7L2* SNP rs7903146; figure reproduced with permission from [23]. To convert glucose values from from mg/dl to mmol/l, please multiply by 0.0555

An exception to this picture emerges with genetic variants in *TCF7L2*, a diabetes risk gene associated with incretin resistance [20]. Glucagon-like peptide-1 (GLP-1) infusion strongly induces insulin secretion in the experimental setting of a hyperglycaemic–euglycaemic clamp and this effect is clearly reduced in carriers of the *TCF7L2* risk allele (Fig. 2a). When the glucose level increases, this gene variant seems to affect the ability of an individual to respond with a compensatory secretion of insulin [23] (Fig. 2b). Figure 2b shows that individuals with the wild-type C allele of the rs7903146 single-nucleotide polymorphism (SNP) and individuals who are heterozygous for this SNP adequately respond to increasing glucose with increasing insulin secretion. In contrast, homozygous carriers of the T allele even show decreased insulin secretion at increasing glucose concentrations. This finding can be explained by reduced incretin signalling in these individuals [42].

This phenomenon has also been described by other groups [43–45]. A recent pharmacogenetic study showed that homozygous T allele carriers of the rs7903146 SNP of *TCF7L2* are partially resistant to therapy with dipeptidyl peptidase-4 (DPP-4) inhibitors, which are known to increase GLP-1 availability [46]. Approximately 10% of individuals in our database are homozygous carriers of this T allele, and the gene variant probably contributes to an inability to upregulate insulin secretion. It is important to note that a reduction in glucose levels through lifestyle intervention can reverse the reduced insulin secretion [23, 47]. Therefore, attempts to lower glucose levels both by lifestyle intervention and by pharmacotherapy might be able to slow down the disease progression in this subgroup of prediabetic individuals. Clinical studies to test this hypothesis are on the way. The role of this gene variant in glucose-induced insulin secretion and glucose metabolism has been addressed in many studies [48–51], some of which suggest that the gene variant affects glucose-induced insulin secretion and the conversion of proinsulin to insulin [38] as well as affecting glucose metabolism [50].

## Body fat composition

### Metabolically healthy and unhealthy obesity

**Fig. 3:**
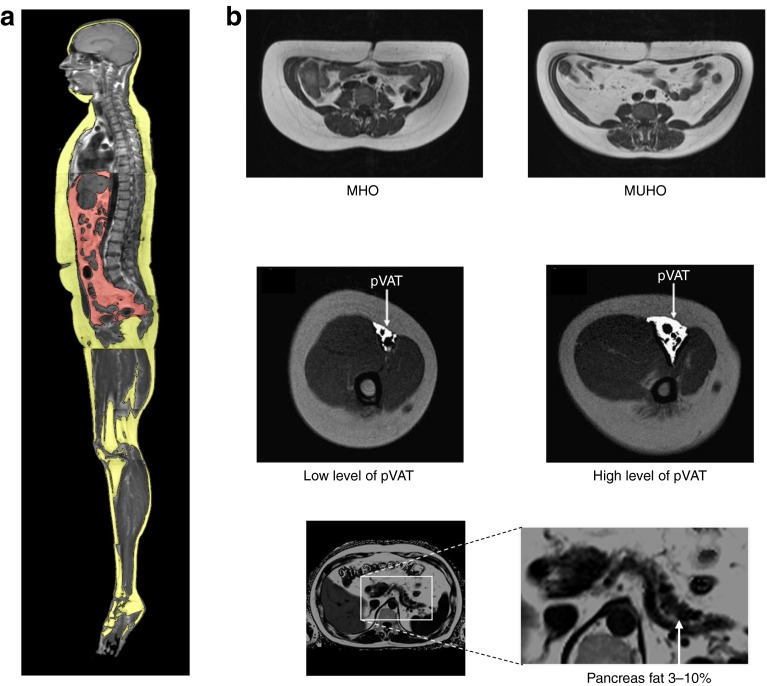
Subphenotypes of obesity. Whole-body MRI measurements are used to quantify fat compartments [52–58]. (**a**) Yellow, subcutaneous adipose tissue; red, visceral adipose tissue. (**b**) pVAT, perivascular visceral adipose tissue

Studies using whole-body MRI not only allow identification of established metabolically relevant fat compartments [52] but also show new fat compartments like neck fat [53] and perivascular fat [54]. Furthermore, magnetic resonance spectroscopy technology allows determination of ectopic fat storage in the liver and the skeletal muscle [52]. A key observation made in such studies has been the description of subphenotypes of obesity: metabolically healthy obesity (MHO) and metabolically unhealthy obesity (MUHO) (Fig. 3).

**Fig. 4:**
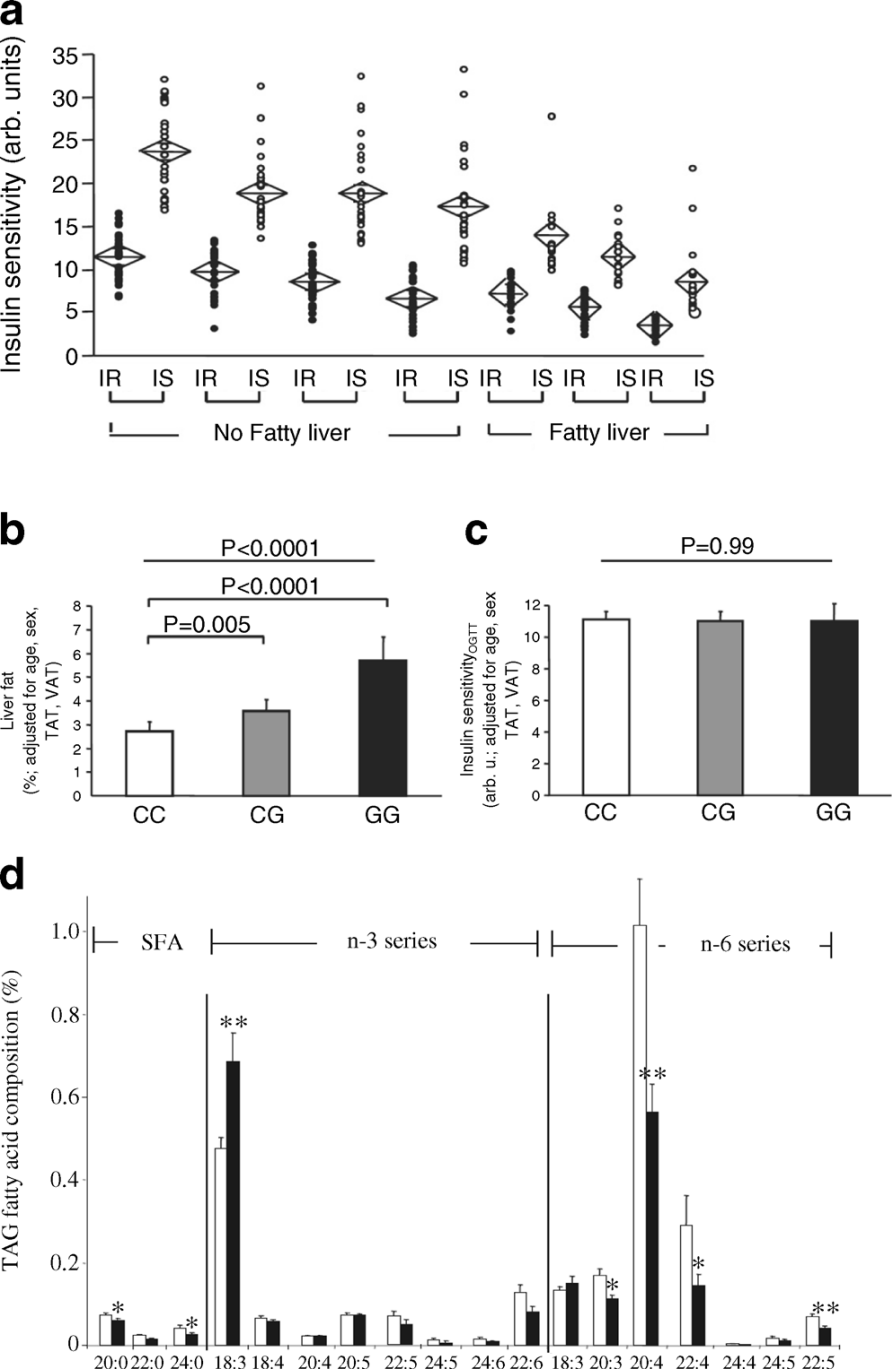
(**a**) Association of insulin resistance with the amount of liver fat. Individuals were divided into seven groups: quartiles of liver fat in individuals without fatty liver (liver fat <5.56%, *n*=225) and tertiles of liver fat in individuals with fatty liver (liver fat ≥5.56%, *n* = 112). Each group was then divided by the median insulin sensitivity into an insulin-sensitive (IS, white circles) and an insulin-resistant (IR, black circles) subgroup. Diamonds indicate mean and the 95% CI. Within each of the seven groups, the subgroups did not differ in liver fat. However, insulin sensitivity was lower in each group; figure reproduced with permission from [58]. (**b**, **c**) Liver fat content (**b**) and insulin sensitivity (**c**) associated with the I148M variant of *PNPLA3*,; figure reproduced with permission from [60]. (**d**) Fatty-acid profiles of hepatic triacylglycerol stores are dependent on the I148M variant of *PNPLA3*. White bars, wild-type individuals; black bars, *PNPLA3* ^I148M^ individuals; figure reproduced with permission from [61]. TAG, triacylglycerol; TAT, total adipose tissue; VAT, visceral adipose tissue

We found that approximately 25% of the obese individuals in our cohort displayed a metabolically healthy phenotype [55–57]. These individuals predominantly accumulate less fat in the liver and store less fat in the visceral compartment, while fat storage in the subcutaneous compartment is high. Furthermore, they display high insulin sensitivity despite having a high BMI [58]. However, most of the obese individuals in our cohort had a metabolically unhealthy phenotype of fat distribution, characterised by decreased subcutaneous fat storage and increased fat storage in the visceral compartment and by non-alcoholic fatty liver disease (NAFLD) (Fig. 3). The insulin resistance of these individuals is associated with the amount of liver fat (Fig. 4a). However, it is evident that at each level of liver fat content a more-insulin-resistant group can be distinguished from a less-insulin-resistant group [57, 58] (Fig. 4a). We saw these data as evidence for distinct liver phenotypes—metabolically benign and metabolically malignant fatty liver [58].

### Genetic predisposition

A genetic predisposition seems to contribute to these phenotypes. Genome-wide association studies have identified a number of genetic variants for NAFLD [59]. The I148M variant of *PNPLA3*, the gene encoding patatin-like phospholipase domain-containing protein 3, is most strongly associated with increased liver fat content (Fig. 4b) [59]. Surprisingly, however, no insulin resistance is observed in individuals possessing this variant (Fig. 4c) [60]. We found that this genotype appears to cause a different fatty-acid composition in the lipid stores of the hepatocytes—decreased levels of stearate and increased levels of polyunsaturated fatty acids (Fig. 4d) [61, 62].

### Role of hepatokines

The different fatty-acid pattern might be responsible for an alteration in the interaction between hepatocytes and the cells of the immune system in the liver. In contrast to the metabolically benign fatty liver, the metabolically malignant fatty liver secretes an altered pattern of hepatokines [63]. The hepatokine fetuin-A is associated with insulin resistance, diabetes and cardiovascular outcomes [63–67]. Saturated fatty acids, such as palmitate, stearate and myristate, have been found to increase hepatic fetuin-A mRNA and protein expression in the human liver cell line HepG2 by increasing NFkB binding to its promoter [68]. Palmitate dose- and time-dependently increases the secretion of fetuin-A from HepG2 cells [68]. Also, high glucose levels dose-dependently increase fetuin-A mRNA and protein expression in HepG2 cells via activation of the extracellular signal-regulated kinase-1/2 (ERK-1–ERK-2) signalling pathway [69]. Finally, preliminary data suggests that exendin-4 may attenuate the expression of fetuin-A in HepG2 cells by improving palmitate-induced endoplasmic reticulum stress through AMP-activated protein kinase [70]. The exact mode of action of fetuin-A is still not fully understood.

George Grunberger’s group was the first to show that fetuin-A inhibits signalling through the insulin receptor [71]) and we and others have confirmed fetuin-A’s inhibitory effect on the insulin receptor tyrosine kinase [reviewed in 63, 64, 72]. Downstream effects on stress kinases and NFkB were observed [72].

Another signalling pathway that is modulated by fetuin-A is the fatty-acid signalling pathway, through the Toll-like receptor. Based on mouse data, it has been proposed that fetuin-A acts as an endogenous ligand of Toll-like receptor 4 to promote lipid-induced insulin resistance [73]. This concept seems to be relevant in humans, as we were able to show that, indeed, circulating fetuin-A levels and NEFA interacted to predict insulin resistance in participants of the TÜF study [64]. These data support the concept that organ crosstalk through hepatokines plays a key role in the pathophysiology of prediabetes.

## Perivascular adipose tissue

Perivascular fat cells seem to be a particularly important target of hepatokines, which might function as transducers of organ crosstalk [54, 74–78]. The whole-body MRI data led us to study another interesting fat compartment—perivascular adipose tissue (Fig. 3). Perivascular adipose tissue is a specific fat depot with impact on organ functions [54, 74–78]. The adipose tissue surrounding arteries seems to have an influence on whole-body insulin sensitivity. This effect is independent of other fat compartments like hepatic fat and visceral fat [54]. Due to the strong effect on whole-body insulin sensitivity, we hypothesised that perivascular adipocytes would have specific characteristics that distinguish them from visceral or subcutaneous fat cells. This is indeed the case, as perivascular fat cells produce and secrete higher quantities of angiogenic factors, cytokines and chemoattractants like monocyte chemoattractant protein-1 (MCP-1) [76]. Furthermore, these fat cells seem to be particularly susceptible to organ crosstalk signals from the fatty liver [72]. The hepatokine fetuin-A stimulates, together with fatty acids, cytokine release and MCP-1 expression in these cells [72].

Fat cells are also found around the renal artery in the hilus of the kidney. As the amount of kidney fat in the hilus correlates with hypertension-inducible albuminuria in individuals with prediabetes [77, 78], we speculate that this fat compartment might be relevant in the pathogenesis of diabetic kidney disease. We further observed that these renal fat cells are particularly responsive to crosstalk signals from the fatty liver (i. e. fetuin-A). Thus, the fatty kidney might be a subphenotype of prediabetes that defines a higher risk of developing kidney disease in the context of MUHO and NAFLD. This is of course at the moment a pure speculation that has to be tested in prospective studies.

## The fatty pancreas: non-alcoholic fatty pancreas disease

**Fig. 5:**
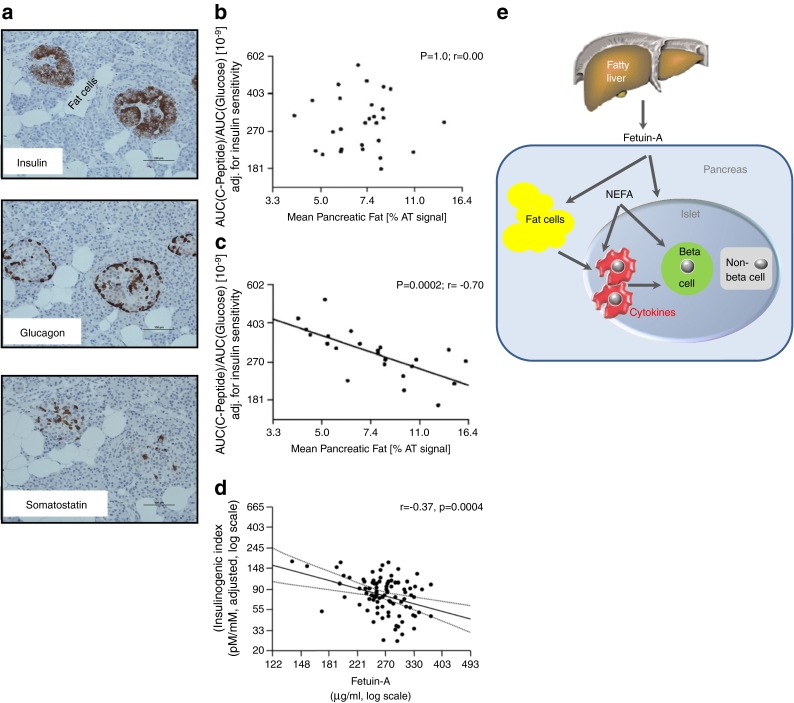
The fatty pancreas. (**a**) Histochemistry of pancreas sections. (**b**, **c**) Association of pancreatic fat and insulin secretion in people with NGT (**b**) and IGT (**c**); figure reproduced with permission from [79]. (**d**) Association of increased fetuin-A from fatty liver with impaired insulin secretion; figure reproduced from [80]. (**e**) Cell-to-cell crosstalk between intrapancreatic fat cells, islets and macrophages

Ectopic fat storage is also observed in the pancreas. In MRI studies, about 3–10% of fat is detected in the pancreas (Fig. 3). The pancreatic fat content does not correlate with insulin secretion in individuals with NGT, although it does correlate strongly with insulin secretion in individuals with IGT [79]. We recently demonstrated that these magnetic resonance-derived fat signals reflect clusters of fat cells in the pancreas, found in close vicinity to islets and sometimes even within islets (Fig. 5a). Macrophages were also detectable. We speculate that fat cells, islets and macrophages are engaged in a cell-to-cell crosstalk (Fig. 5e), which alters the expression of chemoattractants and cytokines. It is likely that this process modifies lipolysis of fat cells and, thereby, fatty-acid signalling. This cell crosstalk is probably silent in the situation of normal glycaemia, while in IGT additional stimulators are present, most likely glucose, cytokines, adipokines and hepatokines. In this respect, we could show that insulin secretion is correlated to the level of circulating fetuin-A in individuals with IGT [80]. Based on this data, we favour the following concept of organ crosstalk in prediabetes: MUHO might induce the development of fatty liver and organ crosstalk, which involves fetuin-A; in the presence of a fatty pancreas, this hepatokine could amplify the cellular crosstalk described in Fig. 5 and this might finally affect islet function and survival and therefore the capacity for compensatory insulin hypersecretion.

## Response to lifestyle intervention

All these observations support a key role for NAFLD in the pathogenesis of insulin resistance and the progression from NGT to IGT and finally to type 2 diabetes. Furthermore, our lifestyle intervention study has clearly shown that lifestyle intervention has limited success in improving glycaemia in prediabetic individuals who have NAFLD [7]. Therefore, other approaches to reduce liver fat content are extremely important.

Dietary intervention is a powerful tool with which to reduce liver fat [81–86]. However, there is evidence that the susceptibility to carbohydrate-dependent induction of liver fat shows a large inter-individual variation [81], suggesting the existence of diet non-responders. A study in patients with biopsy-proven NAFLD showed that a 2 week administration of a very-low-carbohydrate diet (20 g/day) vs energy restriction (5000–6300 kJ/day) reduced hepatic triacylglycerol levels by a greater amount (−55% vs −28%, respectively), while weight loss was similar in both groups (−4.0 kg vs −4.6 kg) [82].

In another study carried out over 16 weeks in 52 individuals with obesity, insulin resistance and suspected NAFLD, a normal carbohydrate (60% carbohydrate, 25% fat, 15% protein) or moderately restricted carbohydrate (40% carbohydrate, 45% fat, 15% protein) diet again resulted in a similar decrease in body weight, daily insulin requirement and plasma liver enzymes levels. However, the moderately restricted carbohydrate intervention was associated with a larger decrease in insulin resistance and liver enzymes [83].

The effect of an isoenergetic diet with restricted fat, restricted saturated fat (LSAT) and restricted glycaemic index (GI) (LSAT: 23% fat [7% saturated fat], GI < 55) on liver fat content was compared with the effect of a high-fat, high-saturated fat (HSAT) and high-GI (HSAT: 43% fat [24% saturated fat] GI > 70) diet in an elderly population. In the LSAT group, but not in the HSAT group, liver fat content decreased significantly [84].

In most studies intake of n-3 polyunsaturated fatty-acid supplements is associated with a reduction in liver fat content, with the doses ranging from 0.83 to 6 g/day, and duration of therapy ranging from 8 weeks to 18 months [85, 86].

The microbiome is probably important as well, although targeted interventions are not feasible as yet.

## The phenotype of non-response to exercise

**Fig. 6:**
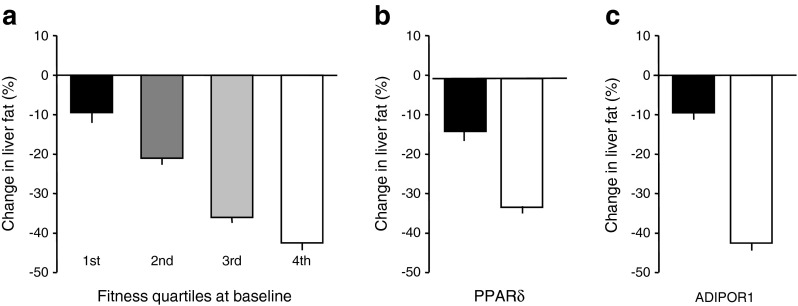
Reduction of liver fat content during a lifestyle intervention is very much related to physical fitness at baseline (*p* = 0.004) (**a**) and to genetic factors (**b**, **c**). Genetic variation in transcription factor PPARδ: black bar, carriers of the risk allele; white bar, non-carriers of the risk allele (*p* = 0.001). Genetic variation in the receptor for adiponectin ADIPOR1: black bar, carriers of the risk allele; white bar, non-carriers of the risk allele (*p* = 0.004). Error bars are SEM. Figures plotted using data from [91] (**a**), [88] (**b**) and [87] (**c**)

From our own experience in the Tübingen lifestyle intervention program (TULIP) study, we suggest that effective reduction of liver fat content during lifestyle intervention is very much related to physical fitness and to variants in the genes for the adiponectin receptor *ADIPOR1* [87] and the transcription factor *PPARδ* (also known as *PPARD*) [6, 88] (Fig. 6).

**Fig. 7:**
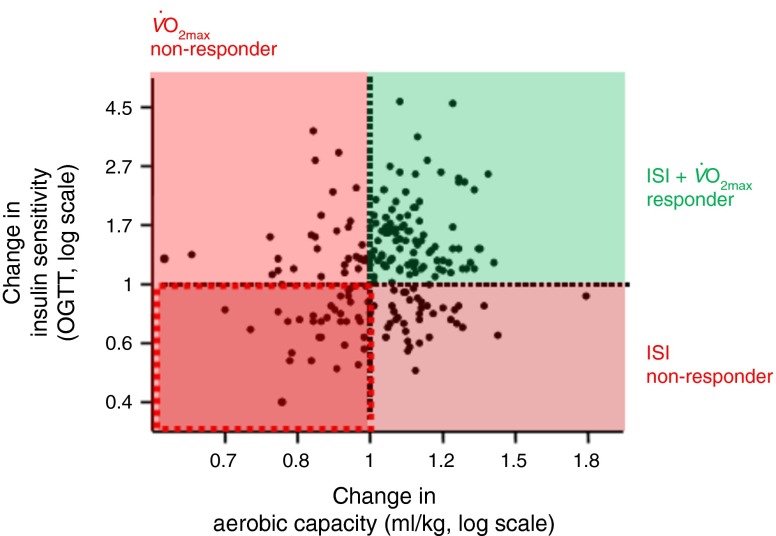
Change in aerobic capacity plotted against change in insulin sensitivity during lifestyle intervention. ISI, Insulin Sensitivity Index calculated from OGTT

Exercise is a key factor in influencing metabolism [89, 90] and reducing liver fat content [91]. However, response to exercise is highly variable. Studies by different groups [90–98; for review see 92] have shown that a certain proportion of prediabetic individuals does not adequately respond to exercise, with respect to fitness variables, like ${\overset{.}{V\mathrm{O}}}_{2 \max }$ and lactate threshold, and insulin sensitivity. We have made the same observation in our lifestyle intervention study (Fig. 7). Understanding the cause of exercise non-response is crucial for improving the success of a lifestyle intervention. We have performed controlled exercise studies in responders and non-responders and have used muscle biopsies to study the underlying molecular mechanisms. It appears that differences in the transcriptional response of muscle cells of the responders and non-responders might explain the different phenotypes as well as differences in the exercise-induced release of myokines [96–98].

As a consequence, differences in the adaptation of fuel oxidation are potentially responsible for these phenotypes.

## Insulin action in the human brain: the phenotype of brain insulin resistance

For a long time the brain was not considered to be a classical target organ of insulin action. However, as early as 1978, Roth and colleagues showed that mouse and rat brain express high levels of insulin receptors [99, 100]. Later, around 2000, several groups showed that alteration of the insulin signalling chain in the brain by knockdown of the insulin receptor or docking proteins causes brain insulin resistance, leading to a diabetes-like phenotype in mice [101–103].

**Fig. 8:**
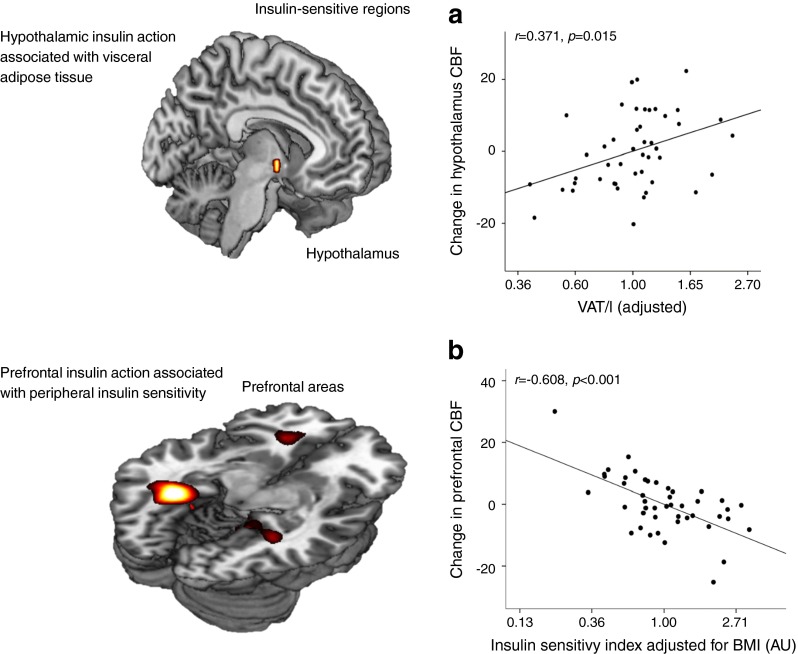
Insulin-sensitive regions in the human brain. (**a**) Hypothalamus. (**b**) Prefrontal area. Data from studies with intranasal insulin application. Figure modified with permission from [106]. CBF, cerebral blood flow; VAT, visceral adipose tissue

For technical reasons it is difficult to demonstrate brain insulin action in humans. We used magneto-encephalography (MEG) to examine insulin effects under the condition of a euglycaemic–hyperinsulinaemic clamp and showed that insulin infusion induces a strong MEG signal in lean individuals but not in obese individuals [104]. We interpreted this as a sign of brain insulin resistance in obese people. In many subsequent studies using functional MRI (fMRI) we further characterised insulin action in the human brain and identified the major insulin-sensitive areas. We have recently reviewed these studies and therein discussed the causes and consequences of brain insulin resistance [105]. Brain insulin signals are detected in the hypothalamus, frontal areas, hippocampus and fusiform gyrus and modulate behavioural functions. We have shown that brain insulin resistance affects very specifically the interaction of the hypothalamus with frontal areas and is associated with insulin sensitivity and amount of visceral fat (Fig. 8) [106]. Very recently, we also showed that brain insulin signalling affects peripheral glucose metabolism [107]. In clamp studies using submaximally active peripheral insulin concentrations, the application of nasal insulin produced an additional increase of glucose uptake in the periphery. Nasal insulin is an efficient tool with which to directly stimulate the human brain [107–111]. This effect of intranasal insulin is closely associated with insulin-dependent activation of the hypothalamus and seems to be transmitted through the autonomous nervous system.

## Brain insulin sensitivity: cause or consequence of adipose tissue distribution?

**Fig. 9:**
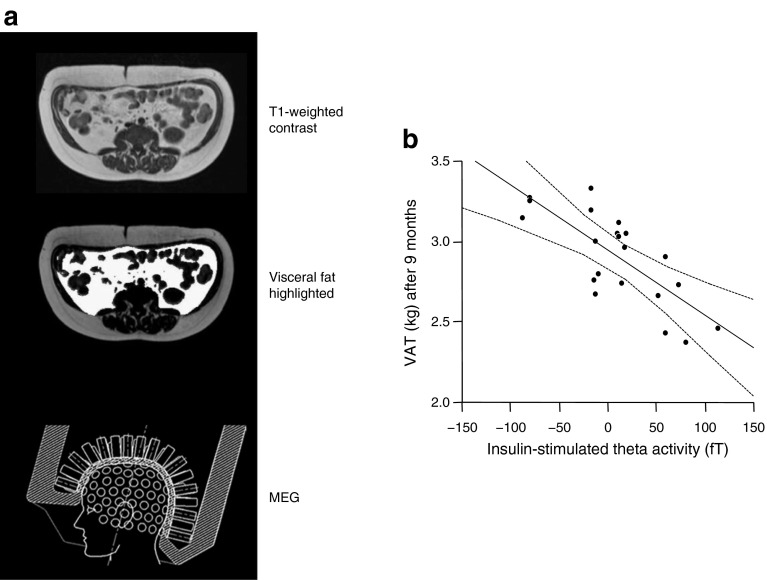
(**a**) Diagrams illustrating the investigation of visceral adipose tissue with magnetic resonance imaging (MRI) and the measurement of cerebral insulin sensitivity using the technique of magnetoencephalography (MEG). (**b**) Brain insulin sensitivity before lifestyle intervention (as insulin-stimulated theta activity) and its association with the change in visceral adipose tissue (adjusted for baseline) during lifestyle intervention; *r* = −0.76; *p* = 0.001; figure reproduced with permission from [112]. VAT, visceral adipose tissue

In our lifestyle intervention study (TULIP) we investigated whether the response to lifestyle intervention is related to brain insulin sensitivity. We found that brain insulin sensitivity was closely related to the change in visceral adipose tissue during lifestyle intervention (Fig. 9) [112]. A potential explanation for this observation might involve autonomous nervous signalling induced by brain insulin action which controls visceral adipose tissue storage. High hypothalamic brain insulin sensitivity associates with increased subcutaneous fat and decreased visceral fat. Low hypothalamic brain insulin sensitivity correlates with high visceral fat and low subcutaneous fat (Fig. 8). This suggests that brain insulin resistance might contribute to the phenotype of MUHO. MHO might depend on strong hypothalamic brain insulin signalling. However, this concept is still very speculative and future studies need to discover whether this hypothesis is valid. While proof from human data is still lacking, several animal models suggest that there is an interaction between brain insulin signalling and the described phenotypes, in particular for fatty liver [113–116]. In accordance with these animal data it has recently been shown that nasal insulin alters fuel flux in the liver in humans [117]. In as yet unpublished studies (M. Heni and A. Fritsche) we recently showed, with the use of stable isotopes, that nasal insulin affects substrate distribution between liver and peripheral tissues. Thus, a crucial role for brain insulin signalling in body fat distribution seems likely.

## Effects of gestational diabetes on fetal brain: does primary brain insulin resistance exist?

Brain insulin resistance can already be found in young obese people [106–111]. This observation allows one to speculate that brain insulin resistance might indeed precede the development of obesity. To further test this hypothesis we studied fetal brain development in pregnancies of insulin-sensitive mothers, insulin-resistant mothers and mothers with gestational diabetes [118, 119]. Fetal brain functions were tested by fetal MEG (fMEG) and the findings suggested that indeed the metabolic situation of the mothers might influence fetal brain insulin sensitivity. It seems therefore conceivable that brain insulin resistance is induced already in utero. Further studies are required to show whether this leads to altered behaviour, altered eating habits and altered weight gain in the postnatal life of these children.

## Brain insulin resistance: starting point for organ crosstalk defining prediabetic phenotypes?

**Fig. 10:**
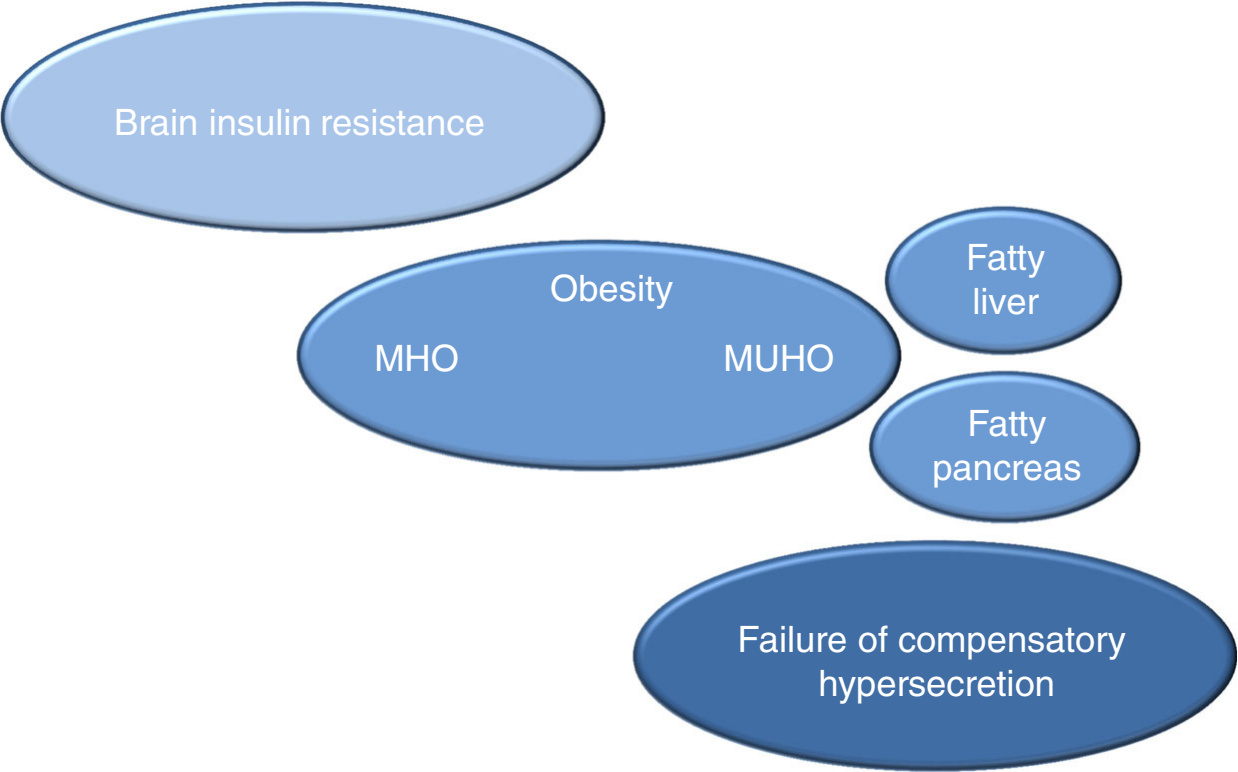
Concept of the pathogenesis of type 2 diabetes mellitus. Chronological development of disadvantageous organ crosstalk involved in the progression from NGT to the prediabetic situation and to type 2 diabetes

Major phenotypes of prediabetic individuals include brain insulin resistance, subphenotypes of obesity (MHO and MUHO), fatty liver, fatty pancreas and variations in perivascular fat. At the level of the pancreas both compensatory insulin hypersecretion and beta cell dysfunction are observed. We speculate that the chronological development of the organ crosstalk is a key feature of the progression from NGT to the prediabetic situation and to type 2 diabetes. Figure 10 illustrates this speculative concept. Brain insulin resistance might occur very early in life and may be the first event contributing to an unfavourable fat distribution pattern (increased visceral fat). MUHO might then be a stepping stone to the development of fatty liver. Hepatokines provide the communication with other organs. Fat cells from perivascular tissue, perihilar fat of the kidney and fat cells in the pancreas seem to be particularly responsive to combined signals of saturated fatty acids and fetuin. These fat cells respond with a pattern of inflammation that probably activates macrophages. The fatty liver might, through these mechanisms, influence both key pathomechanisms of prediabetes, namely insulin resistance and inability to produce compensatory insulin hypersecretion. This scenario is of course very speculative at present but it might be useful as a roadmap for further studies aimed at understanding the chronology underlying the pathophysiology of prediabetes development.

## Abbreviations

GI: Glycaemic index
GLP-1: Glucagon-like peptide-1
HSAT: High saturated fat
IGT: Impaired glucose tolerance
LSAT: Restricted saturated fat
MCP-1: Monocyte chemoattractant protein-1
MEG: Magneto-encephalography
MHO: Metabolically healthy obesity
MUHO: Metabolically unhealthy obesity
NAFLD: Non-alcoholic fatty liver disease
NGT: Normal glucose tolerance
SNP: Single-nucleotide polymorphism
TÜF: Tübingen Family Study
TULIP: Tübingen lifestyle intervention program
